# Dissecting the contribution of microtubule behaviour in adventitious root induction

**DOI:** 10.1093/jxb/erv097

**Published:** 2015-03-18

**Authors:** Mohamad Abu-Abied, Oksana Rogovoy (Stelmakh), Inna Mordehaev, Marina Grumberg, Rivka Elbaum, Geoffrey O. Wasteneys, Einat Sadot

**Affiliations:** ^1^The Institute of Plant Sciences, The Volcani Center, ARO, PO Box 6, Bet-Dagan 50250, Israel; ^2^The Smith Institute of Plant Sciences and Genetics in Agriculture, The Hebrew University of Jerusalem, Rehovot 76100, Israel; ^3^Department of Botany, The University of British Columbia, 6270 University Boulevard, Vancouver, British Columbia V6T 1Z4, Canada

**Keywords:** Adventitious roots, *Arabidopsis*, auxin, cell wall, microtubule, oryzalin.

## Abstract

Induction of adventitious roots (ARs) in recalcitrant plants often culminates in cell division and callus formation rather than root differentiation. Evidence is provided here to suggest that microtubules (MTs) play a role in the shift from cell division to cell differentiation during AR induction. First, it was found that fewer ARs form in the temperature-sensitive mutant *mor1-1*, in which the MT-associated protein MOR1 is mutated, and in *bot1-1*, in which the MT-severing protein katanin is mutated. In the two latter mutants, MT dynamics and form are perturbed. By contrast, the number of ARs increased in *RIC1-OX3* plants, in which MT bundling is enhanced and katanin is activated. In addition, *any1* plants in which cell walls are perturbed made more ARs than wild-type plants. MT perturbations during AR induction in *mor1-1* or in wild-type hypocotyls treated with oryzalin led to the formation of amorphous clusters of cells reminiscent of callus. In these cells a specific pattern of polarized light retardation by the cell walls was lost. PIN1 polarization and auxin maxima were hampered and differentiation of the epidermis was inhibited. It is concluded that a fine-tuned crosstalk between MTs, cell walls, and auxin transport is required for proper AR induction.

## Highlight

Perturbations to microtubules during adventitious root induction lead to the formation of amorphous clusters of cells in which cell walls, coherent auxin transport, and differentiation of root epidermis are disrupted.

## Introduction

Adventitious root (AR) formation helps plants respond to environmental stresses such as water logging. It is also exploited for the propagation of cuttings in agriculture and forestry, so there is intense interest in understanding the mechanisms that drive this complex process of root differentiation and regeneration from non-root tissues (Riov *et al.*, 2013). AR formation in cuttings occurs in four steps, in each of which auxin plays a major role: (I) cell de-differentiation, (II) induction of cell division, (III) development of root primordia, and (IV) root emergence (De Klerk *et al.*, 1999).

In many difficult-to-root plants the transition from step II to step III is inhibited and, instead of root primordia, clusters of cells or callus are formed (Ballester *et al.*, 1999; Greenwood *et al.*, 2001; Levy *et al.*, 2014; Vidal *et al.*, 2003). Whereas a functional root has 15 different types of cells that together coordinate root growth and development (Benfey and Scheres, 2000), the callus formed in recalcitrant species is an amorphous cluster of cells that fails to function as a root.

Microtubules (MTs) are assembled into the spindle apparatus and the phragmoplast to allow plant cells to execute mitosis, meiosis and cytokinesis (Rasmussen *et al.*, 2011; Wasteneys, 2002). MTs also establish distinct cortical arrays in interphase cells, in which the parallel MT array usually matches the orientation of cellulose microfibril deposition (Paredez *et al.*, 2006). This consequently dictates cell shapes (Wasteneys, 2004; Wasteneys and Collings, 2004; Wasteneys and Fujita, 2006). ARs are formed from cells in inner layers, such as the cambium (Blakesley *et al.*, 1991; Fahn, 1990) or pericycle (Falasca and Altmura, 2003). In lateral roots that are also initiated from an inner cell layer of the root, the pericycle, primordial cells push against and influence the cell layers above them in different ways (Swarup *et al.*, 2008; Vermeer *et al.*, 2014). In turn, inhibition of cell layer separation above the lateral root primordia by specifically suppressing auxin signalling in these layers or by suppressing inflorescence deficient in abscission (IDA) abscission signalling, which together should increase pressure on the dividing cells, affects lateral root primordial morphogenesis and differentiation (Kumpf *et al.*, 2013; Lucas *et al.*, 2013; Vermeer *et al.*, 2014). In rice it was shown that the force exerted by AR primordia together with ethylene signalling and reactive oxygen species leads to epidermal programmed cell death, which is necessary for AR emergence (Steffens *et al.*, 2012). It has long been known that MTs respond to mechanical signals (Hardham *et al.*, 1980; Williamson, 1990; Wymer *et al.*, 1996; Zandomeni and Schopfer, 1994). It is now understood that katanin-dependent MT severing is important for the ability of meristematic cells (Uyttewaal *et al.*, 2012) and pavement cells (Sampathkumar *et al.*, 2014) to respond efficiently to mechanical signals. MTs can be affected by changes in tissue-level stress patterns (Hamant *et al.*, 2008; Heisler *et al.*, 2010; Uyttewaal *et al.*, 2012; Wymer *et al.*, 1996), and their directionality matches predicted maximal stress direction that is influenced both by local geometry and growth axis (Burian *et al.*, 2013). MTs are also influenced by cellulose synthesis (Bringmann *et al.*, 2012; Fisher and Cyr, 1998; Himmelspach *et al.*, 2003; Mei *et al.*, 2012; Paredez *et al.*, 2008).

The feedback loop between MTs and cell wall properties appears to be linked to auxin signalling (Landrein and Hamant, 2013). Auxin, by promoting proton pump activity, induces cell wall acidification (Rayle and Cleland, 1992). As a result, the cell wall protein expansin is activated so that cross links in the cell wall matrix are modified, leading to cell wall loosening (Cosgrove, 2005). Auxin has also been shown to trigger demethylesterification of the pectin homogalacturonan and thereby lower cell wall rigidity (Braybrook and Peaucelle, 2013). Vice versa, cellulose synthesis (Feraru *et al.*, 2011), cell wall properties (Braybrook and Peaucelle, 2013; Hamant *et al.*, 2011; Heisler *et al.*, 2010), and external forces (Nakayama *et al.*, 2012) have been shown to affect the localization of the auxin efflux carrier PIN1 in the plasma membrane. In turn, auxin affects MT orientation (Blancaflor and Hasenstein, 1995; Chen *et al.*, 2014; Dhonukshe *et al.*, 2005; Fu *et al.*, 2005; Lin *et al.*, 2013).

Here it is shown that interference with MTs or the cell wall form can either increase auxin-induced AR induction, or lead to callus-like formation instead of ARs. The reciprocal relationships between auxin, MTs, and cell walls during AR formation are discussed.

## Materials and methods

### Materials

All materials were purchased from Sigma (Rehovot, Israel) unless otherwise mentioned. Alexa Fluor-conjugated antibodies were from Molecular Probes (http://www.invitrogen.com/site/us/en/home/brands/Molecular-Probes.html).

### *Arabidopsis* plants, plasmids, and transformation

*Arabidopsis* seeds were germinated and transformed as previously described (Clough and Bent, 1998). Plasmids containing DR5_pro_:venus, which was transfected into *mor1-1* plants, was kindly provided by the Meyerowitz laboratory (Heisler *et al.*, 2005). Other plants used were wild-type *Arabidopsis thaliana* ecotype Columbia or *A. Landsberg erecta*. Mutants with MT-associated protein mutations were *mor1-1* (Whittington *et al.*, 2001) and *rid5* the latter provided by the Sugiyama laboratory (Konishi and Sugiyama, 2003). The DR5_pro_:venus seeds (Laskowski *et al.*, 2008) were kindly provided by Prof. Ben Scheres. Seeds of pGL2::GFP, a root epidermis marker, were provided by Prof. John Schiefelbein from the University of Michigan (Lin and Schiefelbein, 2001).

### Induction of AR formation in *Arabidopsis* plants

ARs were induced in intact plants as previously described (Abu-Abied *et al.*, 2012; Gutierrez *et al.*, 2009; Rasmussen *et al.*, 2012). Briefly, seeds were germinated on Murashige and Skoog (MS) with 0.8% agar plates supplemented with 3% sucrose. The plates were kept in the dark for 2 d at 4°C, then in the dark for 4 d at 22°C, 3 d light, 2 d dark, and 5 d light. After 14 d, ARs (above the collet) were counted. ARs were also induced in cut etiolated hypocotyls that were incubated in MS supplemented with 1% sucrose and 10 μM potassium-indole 3 butyric acid (K-IBA) in the dark. Oryzalin was dissolved in DMSO and applied at 25–300nM. Isoxaben was dissolved in ethanol and applied at 0.1nM, 1nM, or 10nM. Root induction was analysed during the first 3 d and after 6 d. Whole *GL2:GFP* seedlings (7 d old) were incubated in 10 μM K-IBA in the presence or absence of 10nM isoxaben or 100nM oryzalin for 3 d to determine the effect of the treatments on the GFP signal in primary root epidermis. From each treatment 50–70 primordia were scored for GFP in the epidermis.

### Microscopy

Immunostaining was performed as previously described (Chaimovitsh *et al.*, 2011). Images of primordium MTs are projections of several optical sections, 0.5 μm apart, filtered by the rolling ball filter. For MT orientation quantification, the images were analysed by FiberScore (Lichtenstein *et al.*, 2003) and coloured maps of MT orientation were prepared. Cells with parallel and oblique or random MTs were counted manually. From each treatment, 100–250 cells were counted from 7–20 primordia. A representative movie showing the sequential optical sections was also prepared from each treatment. Staining of PIN1 was performed using an antibody kindly provided by J. Friml. Primordia of plants expressing the DR5_pro_:venus were fixed and stained. The venus signal was preserved during fixation. The DR5 signal was measured using FV500 software (Olympus, Hamburg, Germany); the average fluorescence was measured from an equal circle placed on the nuclei, one by one. Each stage V primordium or a cluster of similar size was divided into two: the proximal half closer to the pericycle layer of the hypocotyl and the tip half that is the distal portion. DR5 fluorescence was expressed as the ratio between the average fluorescence of nuclei present in these two halves for each primordium. Measurements included aproximately120 nuclei from five primordia for each treatment. For PIN1 quantification, cells were divided into two groups; those that showed polarized localization of PIN1 at the cell face towards the tip and those that did not. Cells were counted manually and included approximately 200 cells from 7–10 primordia from each treatment.

An Olympus IX81/FV500 laser-scanning microscope was used to observe fluorescently labelled cells with the following filter sets: for enhanced GFP, 488-nm excitation and BA505–525; for Alexa Fluor 555, 543-nm excitation and BA610. The objectives used were PlanApo 60X1.00 WLSM ‘/0.17. When enhanced GFP and Alexa Fluor 555 antibodies were detected in the same sample, dichroic mirror 488/543 was used. In all cases in which more than one colour was monitored, sequential acquisition was performed.

Light retardation was investigated using an LC-PolScope image processing system (CRi, Inc., Woburn, MA, USA) mounted on a microscope (Nikon Eclipse 80i, Tokyo, Japan) equipped with Plan Fluor 920/0.5 OFN25 DIC N2, Plan Fluor 940/0.75 OFN25 DIC M/N2, Fluor 960/100w DIC H/N2 ∞/ 0 WD 2.0 objectives. The system includes a computer-controlled universal compensator made of two liquid crystal variable retarders. Retardation images were taken by a cooled charge-coupled device camera at high optical resolution. Retardation values were extracted manually using Abrio software tools (CRi, Inc.) from 150 to 400 cells from each sample.

### Histological preparations

For light retardation analysis, tissue was fixed in formalin/acetic acid/alcohol fixative (FAA; 50% ethanol, 5% acetic acid and 10% formaldehyde in H_2_O) overnight at room temperature. Tissues were gradually dehydrated with an ethanol series (50%, 70%, 95%, 100%, 100%; 1h each), and then ethanol was gradually replaced by Histo-Clear in five 1-h steps in a ratio of Histo-Clear to ethanol of 1:3, 1:1, and 3:1, with two final steps of pure Histo-Clear. The Histo-Clear was then gradually replaced by paraffin (Paraplast X-TRA). Sections (10 µm) were cut with a rotary microtome (Leica RM2255), deparaffinized, and mounted in Eukitt mounting medium (Kaltek, Padova, Italy) under a coverslip.

## Results

### AR induction rate is affected by mutations in MT-associated proteins

**Fig. 1.**
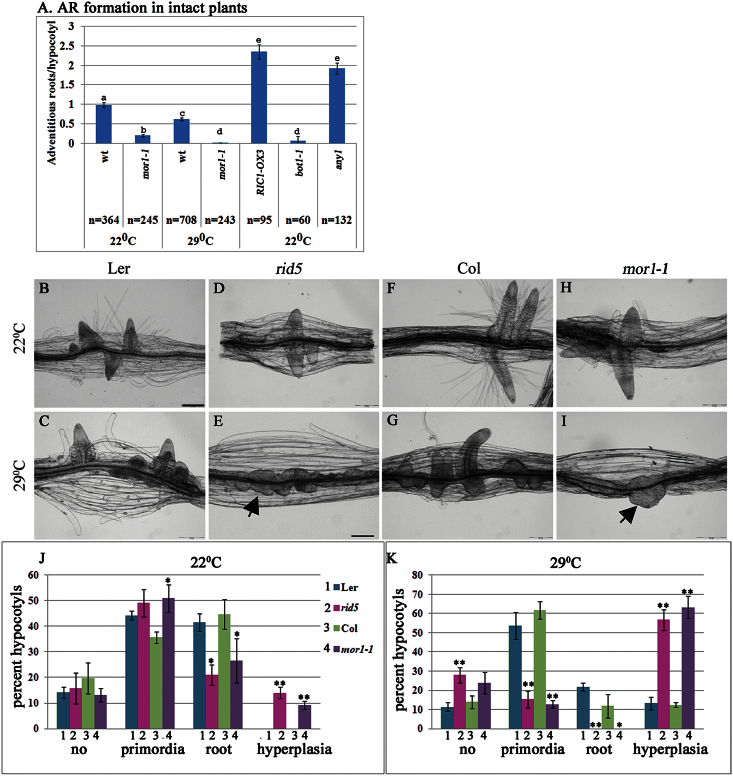
AR formation is affected in plants with disordered MTs or cell walls. (A) Plants overexpressing the ROP effector RIC1 (RIC1-OX3) or carrying mutations in the MT-associated proteins MOR1 (*mor1-1)* and katanin (*bot1-1*) or the cellulose synthase CESA1 (*any1*) were grown in vertical plates and transferred twice from dark to light. After 14 days, ARs were counted. Bars with different letters are significantly different as determined by Scheffe analysis *P* < 0.05; n = number of plants tested. (B-K) Hypocotyls from *mor1-1*, *rid5*, and control wild-type plants (5–6mm in length) were excised from etiolated seedlings and incubated in MS with 1% sucrose and 10 μM K-IBA for 3 d at 22°C (B, D, F, H) and 29°C (C, E, G, I). (J, K) Quantitative analysis of the percentage of hypocotyls without roots (no), with primordia, with roots and with hyperplasia. Scale bars are 200 μm. Asterisks show significant difference from control wild-type plants as determined by Scheffe analysis *P < 0.05, **P < 0.01. Arrows in E and I show amorphous clusters of cells.

In this study, AR formation was induced either in intact seedlings by two sequential transfers from dark to light as previously described (Gutierrez *et al.*, 2009; Rasmussen *et al.*, 2012) or by application of K-IBA to cut etiolated hypocotyls. To investigate the possibility that perturbation of MT dynamics affects ARs, AR formation was followed in various mutant plants. First tested were *rid5* and *mor1-1*, mutant alleles of the *MOR1* gene that encodes an orthologue of the XMAP215 class of MT-associated proteins (Whittington *et al.*, 2001). The mutant *rid5* was isolated in a screen for temperature-sensitive mutants with aberrations in AR formation (Konishi and Sugiyama, 2003), whereas *mor1-1* was identified in a screen for temperature-dependent disruption of MT organization (Whittington *et al.*, 2001). At the restrictive temperature, 29°C, *mor1-1* MTs become short and lose parallel orientation (Whittington *et al.*, 2001) as a result of reduced MT plus-end dynamics (Allard *et al.*, 2010; Kawamura and Wasteneys, 2008). At permissive temperature (22°C), there are subtle but statistically significant reductions in MT plus-end dynamics (Kawamura and Wasteneys, 2008) but these changes have no obvious effect on array organization or plant form (Whittington *et al.*, 2001). AR formation in these plants was induced by twice transferring intact seedlings from dark to light. Compared with wild-type seedlings, *mor1-1* plants produced significantly fewer ARs at permissive temperature (22°C) and almost no ARs at the restrictive (29°C) temperature (Fig. 1A and Fig. S1). This significant reduction in AR formation at permissive temperature suggests that very subtle changes in MT dynamics can affect AR induction despite there being no changes in overall array organization.

To test whether increased auxin concentrations lead to AR induction, cut etiolated *mor1-1* and *rid5* hypocotyls were induced to form ARs in the presence of K-IBA. Fig. 1B shows that in the presence of ectopic auxin, *mor1-1* and *rid5* mutants produced amorphous clusters of cells (hyperplasia) at the restrictive temperature, instead of the dome-like AR primordia seen in wild-type plants or in these mutants at the permissive temperature. To test AR formation in plants with distinct MT alterations, the katanin mutant *bot1-1* (Bichet *et al.*, 2001), was chosen. Fig. 1A and Fig. S1 show less AR formation in this mutant plant. By contrast, plants overexpressing the ROP GTPase effector protein RIC1 (*RIC1-OX3*), in which MT bundle formation (Fu *et al.*, 2005; Fujita *et al.*, 2011) and katanin-mediated MT severing activation (Lin *et al.*, 2013) have been demonstrated, produced more ARs than control wild-type plants (Fig. 1 and Fig. S1). In addition, root induction in *RIC1-OX3* etiolated hypocotyls was less sensitive to oryzalin, an MT-disrupting drug (Fig. S2), suggesting that the excess rooting is related to increased MT stability in these plants. Importantly, mild treatment of wild-type plants with oryzalin during AR induction led to increased formation of amorphous clusters of cells (hyperplasia) (Fig. S3), as in *mor1-1* and *rid*5 plants at 29°C.

**Fig. 2.**
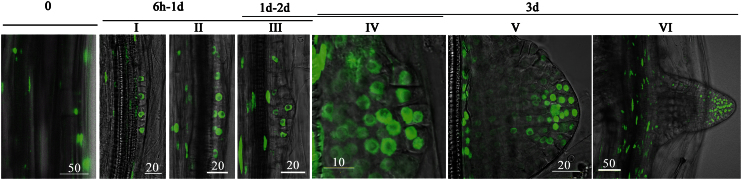
The seven stages of AR formation. Hypocotyls (5–6mm in length) of DR5_pro_:venus-expressing plants were excised from etiolated seedlings and incubated in MS with 1% sucrose and 10 μM K-IBA. Initial stages of AR formation were imaged at the indicated time periods. Scale bars are in nanometres. Stage 0: before root induction. Stage I: four founder cells are formed after anticlinal cell division. Stage II: the first periclinal cell division occurs to create two layers. Stages III, IV and V: more periclinal and anticlinal cell divisions occur to create 3–4, 5–8, and 9–15 cell layers, respectively. Stage V: the primordium acquires the classical dome like shape. Stage VI: root emergence.

For further analysis, six stages of AR development were determined (Fig. 2). Stage 0 is prior to root induction. In stage I, the four founder cells are formed after anticlinal cell division. Stage II comprises the first periclinal cell division to create two layers. During stages III, IV, and V, more periclinal and anticlinal cell divisions occur to create 3–4, 5–9, and 10–15 cell layers, respectively. In stage V the primordium acquires the classical dome shape and stage VI is when the root emerges. All further analyses were done on stage V primordia or cell clusters of similar size.

**Fig. 3.**
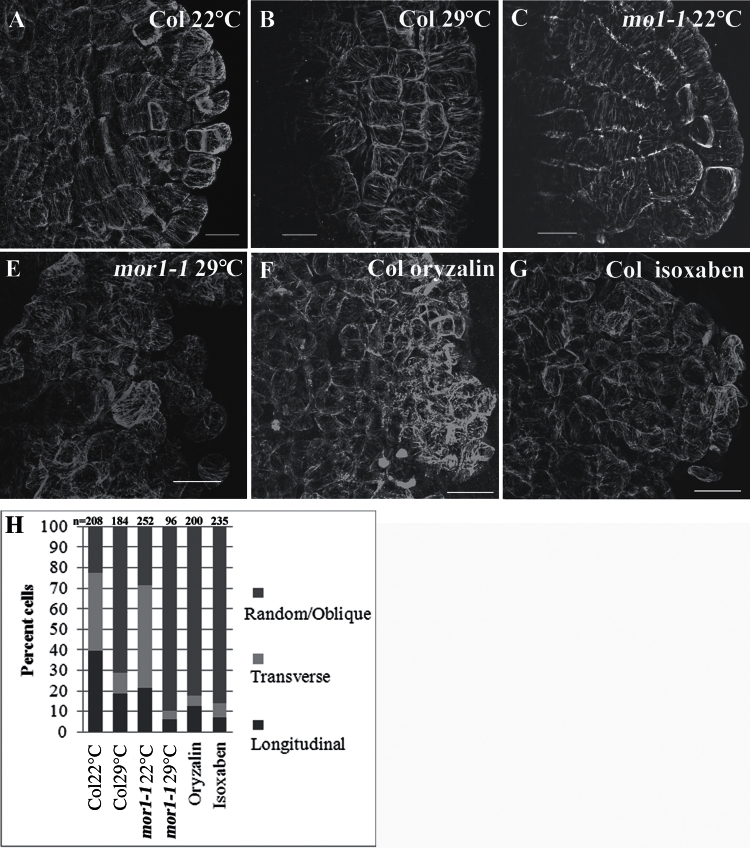
MTs in ARs of (A-E) *mor1-1* and wild-type (Col) plants at permissive or restrictive temperatures, or in (F) Col plants treated with 100nM oryzalin or (G) 10nM isoxaben. Cut etiolated hypocotyls were treated with K-IBA and fixed and stained for MT after 3 d. Images are projections of several optical sections, after filtration by the rolling ball filter. Orientation of MTs was analysed by FiberScore (Fig. S1). (H) Numbers of cells containing the different MTs were counted manually. Scale bars are 10 μm in A-C and 20 μm in D-F.

An immunolabelling method using GFP-tubulin expressing plants was used to document MT patterns because it is difficult to follow MTs in live root primordial. MTs were stained in stage V AR primordia and found to be organized in parallel arrays in wild-type and *mor1-1* plants at 22°C. In wild-type plants at 29°C there was a reduction in the number of cells with transverse or longitudinal parallel arrays. In *mor1-1* plants at 29°C and in wild-type plants after treatment with oryzalin or the cellulose synthesis inhibitor isoxaben, MTs lost parallel order and became randomly oriented or oblique in most cells (Fig. 3, Fig. S4, and Movies S1-6). Of note, previous studies have found hyper-alignment of MTs after short-term (a few hours) isoxaben treatment (Heisler *et al.*, 2010; Paredez *et al.*, 2008; Sampathkumar *et al.*, 2014). By contrast in this study, long-term isoxaben treatment was applied and MTs were followed in cell clusters that formed in its presence over 3 d.

### Accurate cell wall properties are important for AR induction

To determine if the properties of cell walls are involved in proper AR formation, AR induction was performed in *any1* plants, which have a missense mutation in the CESA1 cellulose synthase. These plants have reduced anisotropic growth and reduced cell wall crystallinity (Fujita *et al.*, 2013). More ARs were formed in this mutant compared to wild-type plants (Fig. 1 and Fig. S1). In contrast, when wild-type hypocotyls were treated with a constant amount of K-IBA and increasing concentrations of the cellulose synthesis inhibitor isoxaben, AR formation was inhibited in a dose-dependent manner (Fig. S5). Interestingly, after 3 d of treatment with 0.1nM, 1nM, or 10nM isoxaben, 38%, 42%, and 80% of the hypocotyls, respectively, exhibited amorphous clusters of cells (hyperplasia), showing that cell division continues but cell differentiation is hampered. More roots formed after 6 d, suggesting that in some cases differentiation was inhibited but not abolished.

**Fig. 4.**
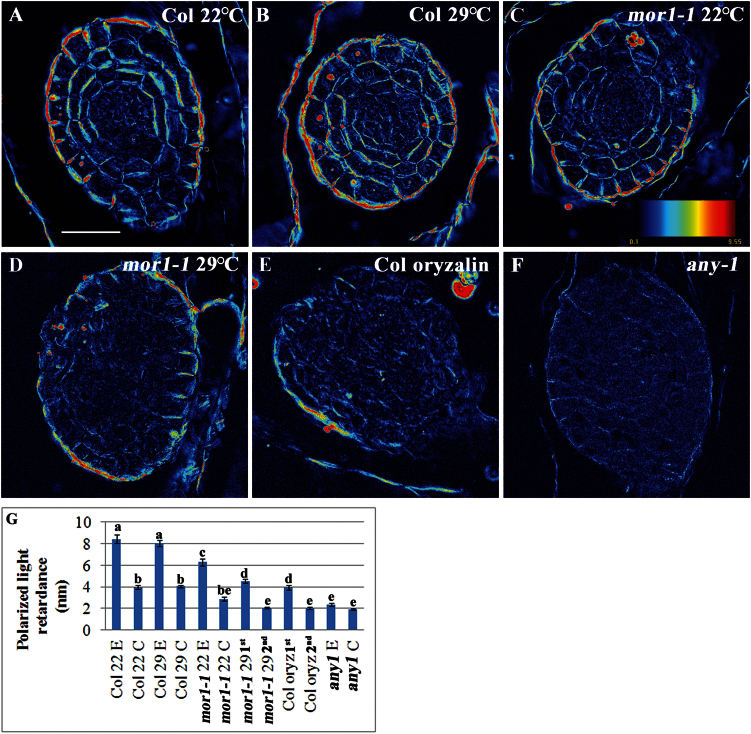
The wall properties of cells of AR primordia and of the amorphous clusters that form when MTs are perturbed. Etiolated hypocotyls of (A-B) wild-type (Col) or (C-D) *mor1-1* plants were induced to form ARs at 22°C or 29°C. Col plants were also treated with (E) oryzalin (100nM). (F) As a control for the sensitivity of the method to changes in cell walls, *any1* plants were used. After 3 d in the dark in the presence of K-IBA, the hypocotyls were fixed, embedded in paraffin, and dissected by a rotary microtome. The sections were deparaffinized, mounted between two microscope slides, and imaged by an LC PoleScope. Light retardation was measured from the outer cell wall of all epidermal (E) and cortex (C) cells of 4–10 primordia or from the first and second layers in cell clusters. The coloured scale is from blue (0nm) to red (10nm) wave lengths. The average perimeter of all primordia and clusters measured was 80–90 μm. (G) The graph shows averages of light retardation ± standard error. Bars with different letters are significantly different as determined by Scheffe analysis *P* < 0.001. Scale bar is 30 μm.

To further examine the effect of MTs on the walls of AR primordia cells, polarized light retardation was determined and quantified using an LC PolScope (Abraham and Elbaum, 2013). It was first determined that the signal obtained by the polarized light shows parallel arrays in stage V primordium epidermal cells similar to that of MTs (Fig. S6). Next, serial sections of single primordia were examined to determine whether xylem typical cell wall thickenings, rich in crystalline cellulose, are present in stage V primordia. Fig. S7 shows high light retardation by the xylem of the hypocotyl but no such signal in the AR primordia. Light retardation was then compared in transverse sections of stage V primordia or cell clusters of similar size. In wild-type plants, a higher retardation was observed in the outer face of epidermal cells and a circumferential gradient was found from the epidermis inward, showing reduced retardation at the outer face of cells in inner layers (Fig. 4). This pattern indicates specific polarity of cell wall architecture at the cellular level along with a specific gradient and radial symmetry at the tissue level, which are characteristic for differentiated root primordia. This pattern was hampered in wild-type plants treated with oryzalin and in *mor1-1* plants at 29°C. As a control, *any1* plants, in which cellulose organization is changed and crystallinity is reduced, were used. A dramatic reduction in light retardation was observed in *any1* AR primordial cells (Fig. 4). It is concluded that MTs are important for optimal cell wall formation during AR differentiation, but when MTs arrays and dynamics are intact, normal AR primordia are formed even in the presence of slight perturbations to the cell wall.

### The amorphous clusters of cells exhibit non-polarized PIN1, lack of auxin maxima, and failure of epidermis differentiation

**Fig. 5.**
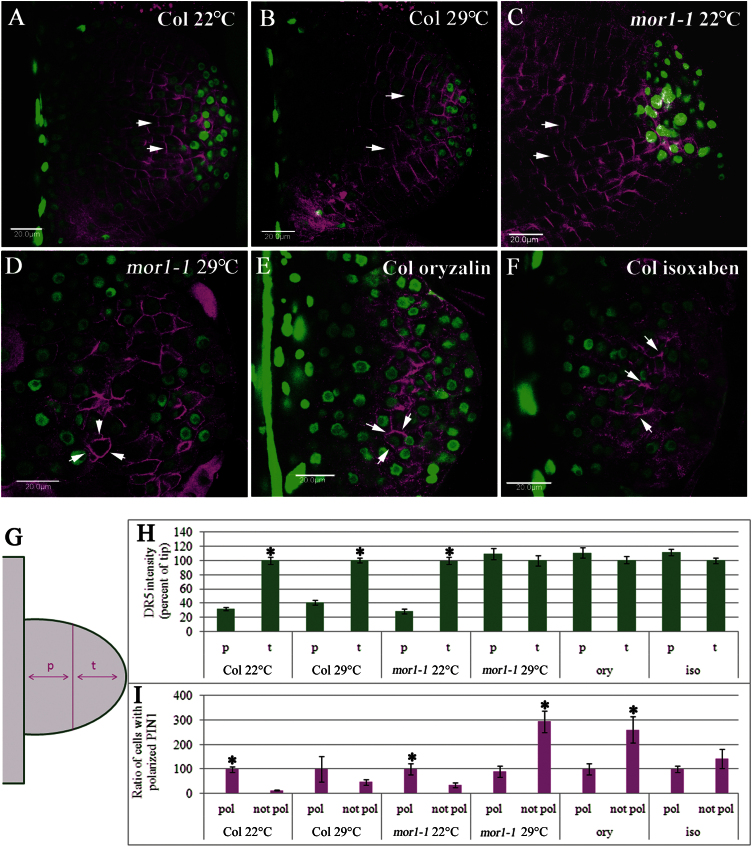
AR formation in *mor1-1* and wild-type plants expressing the DR5_pro_:venus marker of auxin activity. (A-F) Three days after root induction with auxin, the hypocotyls were fixed and stained for PIN1 (magenta). (G) For quantitative analysis, each primordium was divided into the proximal half (p) and the tip half (t). (H) The fluorescence intensity of GFP was measured separately for each nucleus by the FV500 software. For each primordium, the values were expressed as a percentage of the average fluorescence calculated for the nuclei in the tip. (I) Cells with PIN1 either polarized to the cell face towards the AR primordial axis (pol) or found in more than one cell face (not pol) were counted. Bars represent averages ± standard error. Asterisks mark significantly different bars as determined by Scheffe analysis *P* < 0.01 Scale bars are 20 μm.

To address the question of how auxin accumulates in the amorphous clusters of cells formed when MTs are perturbed, DR5_pro_:venus (Heisler *et al.*, 2005) was introduced to *mor1-1* plants, which were then compared to wild-type plants (Laskowski *et al.*, 2008). In addition, localization of PIN1 was determined by immunofluorescence. At 22°C, PIN1 was localized in a polar manner to the cell membrane facing the root growth axis, and auxin activity was greatest at the tip of root primordia in *mor1-1*/DR5_pro_:venus as in control plants (Fig. 5). At 29°C, however, polar localization of neither PIN1 nor auxin maxima was observed in the amorphous clusters of dividing cells in *mor1-1*/DR5_pro_:venus plants (Fig. 5). By contrast, polar localization of PIN1 and auxin gradients eventually developed in wild-type plants expressing DR5_pro_:venus, although the formation of root primordia was slightly inhibited by the high temperature (Fig. 5). In the presence of oryzalin, PIN1 lost polarization, and was often distributed to two to four cell sides. In the presence of isoxaben, PIN1 lacked coherent distribution between cells, and within individual cells could be found at more than one face, in the cytoplasm, or, rarely, focused at three-way cell junctions (Fig. 5 and Fig. S8). This is reminiscent of previous findings showing that oryzalin and isoxaben change the distribution of PIN1 in the membrane of shoot apical meristem cells (Hamant *et al.*, 2011), and in line with a previous suggestion that MTs contribute indirectly to PIN1 localization (Boutte *et al.*, 2006; Heisler *et al.*, 2010; Kleine-Vehn *et al.*, 2008). Concomitantly, no auxin maxima were observed in cell clusters developed under slight MT perturbation.

**Fig. 6.**
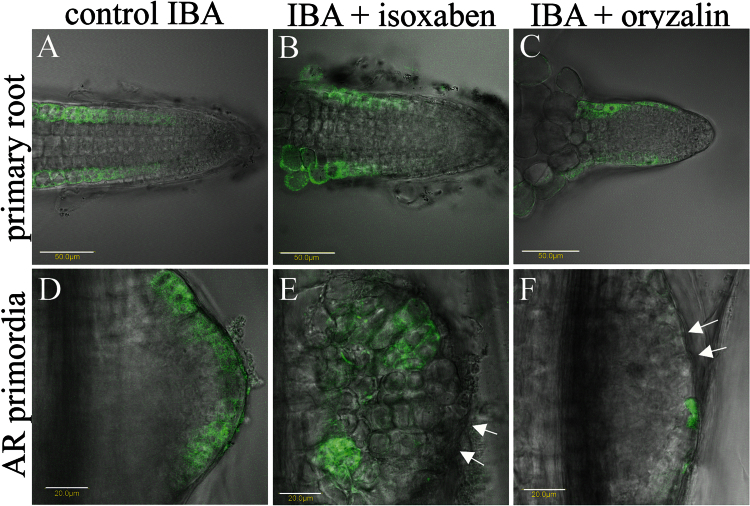
Differentiation of primordia epidermis in control or in oryzalin- or isoxaben-treated plants. Etiolated hypocotyls of *GL2*_*pro*_ *:GFP* plants were induced to form ARs with auxin in the presence of oryzalin or isoxaben. From each treatment, 50–70 primordia were scored for the presence of GFP in the epidermis. **(A-C)** As a control, primary roots were followed under the same conditions. **(D-F)** *GL2*_*pro*_ *:GFP* expression in AR primordia under the different treatments. Scale bars in A-C 50 μm and in D-F 20 μm.

To determine the differentiation status of these clusters, plants expressing the *GL2*_*pro*_ *:GFP* reporter gene were used. This line expresses GFP under the regulation of the promoter of GLABRA2, a transcription factor specific for root epidermal cells (Lin and Schiefelbein, 2001) and therefore an excellent marker of root epidermal identity. It was first confirmed that the epidermis of the primary root preserves the expression of *GL2*_*pro*_ *:GFP* under the experimental conditions (Fig. 6). GFP expression was observed in the epidermis of the primary root following incubation for 3 d in K-IBA in the absence or presence of oryzalin or isoxaben. When etiolated hypocotyls of the *GL2*_*pro*_ *:GFP* plants were induced to form ARs with K-IBA, cells of the outer cell layer expressed the GFP at early stages (IV–VI) in 90% of primordia observed (Fig. 6 and Fig. S9). In the presence of 10nM isoxaben, 70% of the cell clusters did not express GFP; in 30% of the cases only some cells at the outer layer expressed the GFP (Fig. 6 and Fig. S9). In the presence of 100nM oryzalin, only very seldom (1% of the samples) did one or two cells show GFP expression (Fig. 6 and Fig. S9). This confirmed that proper MT organization is critical for root epidermal differentiation during AR formation.

## Discussion

In 1978 Oppenoorth noticed that colchicine, an MT-disrupting drug, inhibited cell de-differentiation and differentiation during AR formation in herbaceous cuttings (petioles) of *Phaseolus vulgaris* (Oppenoorth, 1978). By contrast, in mature woody recalcitrant cuttings of *Eucalyptus grandis*, MT perturbation with low concentrations of trifluralin, another MT-disrupting drug, resulted in increased AR formation (Abu-Abied *et al.*, 2014). This suggests that optimal organization and dynamics of MTs are required for proper AR organogenesis. Therefore, intervening with MTs that might not be in an optimal form for AR formation in recalcitrant plants could increase rooting (Abu-Abied *et al.*, 2014), but intervening with MTs in easy-to-root plants may inhibit rooting (Oppenoorth, 1978).

There is a difference in the pattern of differentiation of lateral organs from roots versus shoots. In roots there is de-differentiation before organogenesis, whereas in shoots there is no dedifferentiation before organogenesis; in roots it is the inner layers driving organogenesis, whereas in shoots it is the outer layers (Oppenoorth, 1978; Murray *et al.*, 2012). Despite these differences, it seems that the crosstalk between MTs, cell walls, and auxin signalling play a role in the induction of lateral outgrowth in both tissues. Local application of oryzalin on *pin1-1* shoot apical meristem promoted outgrowth similar to the effect of local application of indoleacetic acid (Sassi *et al.*, 2014). In another case, Hernandez and Green reported that mechanical signals affect organogenesis. They showed that ectopic pressure changed the organogenesis of *Helianthus annuus* (sunflower) dyad florets, resulting in the formation of one big bract instead of a flower and a bract (Hernandez and Green, 1993). Preferential lateral root formation at the outer edge of root bends (Ditengou *et al.*, 2008; Laskowski *et al.*, 2008; Richter *et al.*, 2009) also suggest the participation of mechanical signals in lateral root induction.

In line with the above, the data presented here suggest that MT organization is important for proper AR induction in *Arabidopsis* plants. While the forces were not measured, the results consolidate the idea that crosstalk between MTs, cell wall properties, and auxin transport is important for the shift from cell division to cell differentiation during AR formation in *Arabidopsis*. It is difficult to define the status of the cells in the clusters that formed when AR were induced in a background of MT perturbations. On the one hand, these clusters are reminiscent of callus formed in recalcitrant plants, but on the other hand it has previously been shown that callus cells express root markers (Sugimoto *et al.*, 2010), but the clusters described here did not express the root epidermal marker GL2pro:GFP. Therefore, it is not clear which particular stage of AR organogenesis is hampered when MTs are perturbed. Oppenoorth proposed that, in the presence of colchicine, clusters of fewer than 30 meristematic cells could not continue to form root primordia (Oppenoorth, 1978). It is proposed that the amorphous clusters found here are in a more or less similar status; these are meristematic cells that do not form root primordia.

### AR induction in *rid5*, *mor1-1*, *bot1-1*, *RIC1-OX3*, and *any1* plants

Intact *mor1-1* plants, when stimulated to produce ARs by transfer from dark to light, generated fewer ARs at the restrictive temperature of 29°C but, interestingly, also at the permissive temperature. This finding is intriguing because although *mor1-1* MT organization patterns and plant growth are indistinguishable from those of wild-type plants grown at the same ‘permissive’ temperature, there are subtle reductions in *mor1-1* MT growth and shrinkage rates (Kawamura and Wasteneys, 2008). This suggests that sustained MT dynamics optimize AR induction and that the failure of ARs to form is not simply a consequence of disrupted MT arrays or altered division patterns. The relationship between MT dynamics and AR induction, however, is complex. On the one hand, a reduction was found in the rate of AR induction at a high temperature (29°C) compared to 22°C, and it has been shown that MT growth and shrinkage rates increase dramatically at higher temperatures (Kawamura and Wasteneys, 2008). On the other hand, higher activity of the auxin-responsive *DR5* promoter was found in *Arabidopsis* seedlings exposed to 29°C (Stavang *et al.*, 2009) but PIN1 density in the plasma membrane of cells at the shoot apical meristem decreased at 37°C (Nakayama *et al.*, 2012). Taken together, it cannot be ruled out that the lower efficiency in AR induction in both wild-type and *mor1-1* plants at the restrictive temperature involves factors unrelated to MT integrity. The level of auxin is likely to be important because the application of K-IBA to cut etiolated hypocotyls led to somewhat similar AR primordia formation in *mor1-1* or *rid5* compared to wild-type plants at 22°C. This is in agreement with the observation that the *rid5* mutant requires higher concentrations of auxin than wild-type plants in order to make roots (Konishi and Sugiyama, 2003).

Plants with katanin loss-of-function mutations also made fewer ARs than wild-type plants. Katanin is a severing protein that is essential for cortical MT remodelling (Lindeboom *et al.*, 2013; Zhang *et al.*, 2013) and the disappearance of peri-nuclear MTs after cell division (Burk *et al.*, 2001). However, it is not clear yet at which step AR formation is inhibited when katanin is not functioning. Interestingly, while a katanin mutant *bot1-1* produced fewer AR than wild-type plants, another katanin mutant, *bot1-7*, produced more flowers than wild-type plants when auxin transport was blocked (Sassi *et al.*, 2014). In addition, while overexpression of RIC1 promoted more AR formation than in wild-type plants, *ric1* plants produced more flowers than wild-type plants under auxin transport inhibition (Sassi *et al.*, 2014). This suggests that MT perturbations can have differential consequences on organogenesis depending on different contexts of auxin and mechanical signalling and on tissue-, cell layer-, and developmental-specific gene expression.

The increased induction of ARs in *RIC1-OX3* plants is consistent with RIC1’s involvement in katanin activation (Lin *et al.*, 2013). RIC1 is a target of the plant Rho-GTPases ROP1 and ROP2/4. ROP2/4 activity inhibits RIC1 binding to MTs and, in turn, RIC1 promotes the formation of MT bundles to inhibit ROP2 from interacting with its effector RIC4 (Fu *et al.*, 2005). RIC4, via the accumulation of cortical actin microfilaments, inhibits PIN1 endocytosis (Nagawa *et al.*, 2012). These findings, along with the observation from this study that the *RIC1-OX3* line is more resistant to oryzalin inhibition of AR formation, suggest that RIC1 promotes AR formation through its effects on MT polymer formation. RIC1 might also be situated in a road junction between MTs and auxin—it is an effector of the GTPase ROP6, and together they are involved in PIN1 and PIN2 internalization (Chen *et al.*, 2012; Lin *et al.*, 2012) and, in turn, are activated by auxin (Xu *et al.*, 2014). Of note, it was previously shown that the crystallinity in cell walls of *RIC1-OX3* plants was lower than that of wild-type plants at 22°C, whereas wall crystallinity in *mor1-1* at 29°C was higher than that of wild-type plants (Fujita *et al.*, 2011). This suggests a causative correlation between low crystallinity and high AR induction. In line with this, *any1* plants, which have reduced cellulose crystallinity (Fujita *et al.*, 2013), made more AR. Nevertheless, cellulose crystallinity remains to be determined in the specific tissue giving rise to ARs.

Cellulose microfibrils were disorganized in epidermal cells of the *any1* inflorescence stems but in the cortex and pith cells no differences from wild-type plants were observed (Fujita *et al.*, 2013). By contrast, light retardation was reduced in both epidermal and cortex cells of *any1* AR primordia compared to wild-type plants. While microfibrils of *mor1-1* root epidermal cells remained predominantly transverse to the elongation axis after MT disruption at 29°C (Sugimoto *et al.*, 2003), a significant decrease in light retardation was found in AR primordia in *mor1-1* at 29°C. A decrease in light retardation was also observed in the amorphous clusters formed when MTs were perturbed by oryzalin. This is in agreement with previous observations in which oryzalin treatment decreased microfibril uniformity in root cells (Baskin *et al.*, 2004). Because cellulose is a birefringent material, polarized light retardation is correlated with cellulose microfibril direction and crystallinity, which in turn affect the rigidity of cell walls (Abraham and Elbaum, 2013). The increased AR formation in *any1* plants is reminiscent of other observations in which softer cell walls favour organogenesis (Braybrook and Peaucelle, 2013; Kierzkowski *et al.*, 2012; Peaucelle *et al.*, 2011; Pien *et al.*, 2001). The decrease in light retardation in the amorphous clusters of cells formed when MTs are perturbed suggests that MTs participate in organogenesis by optimizing cell wall properties, and that changes in cell wall properties in the background of unfavourable MT form might lead to callus-like tissue formation.

### MTs during AR organogenesis

A molecular mechanism that underlies a crosstalk between MTs and auxin transport was recently revealed. CLASP, an MT-binding protein (Ambrose *et al.*, 2007; Ambrose and Wasteneys, 2008; Kirik *et al.*, 2007) accumulates at sharp cell edges and facilitates the interconnection of MTs on different cell faces (Ambrose *et al.*, 2011). Through a direct interaction with the retromer component sorting Nexin 1, CLASP tethers PIN2-containing endosomes to MTs, which promotes recycling and concentration of PIN2 in cells, thereby promoting auxin polar transport (Ambrose *et al.*, 2013). Interestingly, *clasp* plants exhibit auxin-related phenotypes including abundant lateral roots (Ambrose *et al.*, 2007; Kirik *et al.*, 2007) and the formation of callus on etiolated hypocotyls (Ambrose *et al.*, 2013). The role of this mechanism in AR formation is still to be determined.

It is therefore concluded that induction of AR formation when MT dynamics or cell wall properties are not within a narrow optimal range results in increased formation of clusters of dividing cells but a decrease in root differentiation. This may result from disruption of formative cell division (De Smet and Beeckman, 2011) or a disruption of the mechano-sensing machinery (Besnard *et al.*, 2011) or both. Further studies are required to reveal mechano- and/or auxin-sensitive MT-associated proteins, the expression of which is critical for AR formation.

## Supplementary data

Supplementary data are available at *JXB* online.

Figure S1. The different mutant plants that were induced to form AR by the dark-to-light regime.

Figure S2. RIC1-OX3 plants are less sensitive to oryzalin than wild-type plants in terms of AR formation.

Figure S3. AR formation in the presence of low concentrations of oryzalin.

Figure S4. MTs in AR primordial cells.

Figure S5. AR formation in the presence of increasing amounts of isoxaben.

Figure S6. A comparison of MT orientation and polarized light retardation pattern in epidermal cells of AR primordia.

Figure S7. Serial 10 μm sections through an AR primordia, imaged by the PoleScope.

Figure S8. Close-up on the distribution of PIN1 in the presence of oryzalin or isoxaben.

Figure S9. Various stages of AR primordia of GL2_pro_:GFP plants after oryzalin or isoxaben treatments.

Movie S1. MTs in stage V AR primordium of wild-type plants treated with K-IBA at 22°C.

Movie S2. MTs in stage V AR primordium of wild-type plants treated with K-IBA at 29°C.

Movie S3. MTs in stage V AR primordium of *mor1-1* plants treated with K-IBA at 22°C.

Movie S4. MTs in a cell cluster of *mor1-1* plants treated with IBA at 29°C.

Movie S5. MTs in a cell cluster of wild-type plants treated with IBA and oryzalin.

Movie S6. MTs in a cell cluster of wild-type plants treated with IBA and isoxaben.

Supplementary Data

## Abbreviations:

AR: adventitious root
GFP: green fluorescent protein
K-IBA: potassium-indole 3 butyric acid
MT: microtubule.

## References

[CIT0001] Abraham Y Elbaum R 2013 Quantification of microfibril angle in secondary cell walls at subcellular resolution by means of polarized light microscopy. New Phytologist 197, 1012–1019. 23240639 10.1111/nph.12070

[CIT0002] Abu-Abied M Szwerdszarf D Mordehaev I 2012 Microarray analysis revealed upregulation of nitrate reductase in juvenile cuttings of *Eucalyptus grandis*, which correlated with increased nitric oxide production and adventitious root formation. The Plant Journal 71, 787–799. 22519851 10.1111/j.1365-313X.2012.05032.x

[CIT0003] Abu-Abied M Szwerdszarf D Mordehaev I Yaniv Y Levinkron S Rubinstein M Riov J Ophir R Sadot E 2014 Gene expression profiling in juvenile and mature cuttings of *Eucalyptus grandis* reveals the importance of microtubule remodeling during adventitious root formation. BMC Genomics 15, 826. 25266376 10.1186/1471-2164-15-826 PMC4190485

[CIT0004] Allard JF Wasteneys GO Cytrynbaum EN 2010 Mechanisms of self-organization of cortical microtubules in plants revealed by computational simulations. Molecular Biology of the Cell 21, 278–286. 19910489 10.1091/mbc.E09-07-0579 PMC2808237

[CIT0005] Ambrose C Allard JF Cytrynbaum EN Wasteneys GO 2011 A CLASP-modulated cell edge barrier mechanism drives cell-wide cortical microtubule organization in *Arabidopsis* . Nature Communication 2, 430. 10.1038/ncomms1444 PMC3265373 21847104

[CIT0006] Ambrose C Ruan Y Gardiner J Tamblyn LM Catching A Kirik V Marc J Overall R Wasteneys GO 2013 CLASP interacts with sorting nexin 1 to link microtubules and auxin transport via PIN2 recycling in *Arabidopsis thaliana* . Developmental Cell 24, 649–659. 23477787 10.1016/j.devcel.2013.02.007

[CIT0007] Ambrose JC Shoji T Kotzer AM Pighin JA Wasteneys GO 2007 The *Arabidopsis* CLASP gene encodes a microtubule-associated protein involved in cell expansion and division. Plant Cell 19, 2763–2775. 17873093 10.1105/tpc.107.053777 PMC2048705

[CIT0008] Ambrose JC Wasteneys GO 2008 CLASP modulates microtubule–cortex interaction during self-organization of acentrosomal microtubules. Molecular Biology of the Cell 19, 4730–4737. 18716054 10.1091/mbc.E08-06-0665 PMC2575154

[CIT0009] Ballester A San-Jose MC Vidal N Fernandez-Lorenzo JL Vieitez AM 1999 Anatomical and biochemical events during *in vitro* rooting of microcuttings from juvenile and mature phases of chestnut. Annals of Botany 83, 619–629.

[CIT0010] Baskin TI Beemster GT Judy-March JE Marga F 2004 Disorganization of cortical microtubules stimulates tangential expansion and reduces the uniformity of cellulose microfibril alignment among cells in the root of *Arabidopsis* . Plant Physiology 135, 2279–2290. 15299138 10.1104/pp.104.040493 PMC520797

[CIT0011] Benfey PN Scheres B 2000 Root development. Current Biology 10, R813–815. 11102819 10.1016/s0960-9822(00)00814-9

[CIT0012] Besnard F Vernoux T Hamant O 2011 Organogenesis from stem cells in planta: multiple feedback loops integrating molecular and mechanical signals. Cellular and Molecular Life Science 68, 2885–2906. 10.1007/s00018-011-0732-4 PMC11115100 21655916

[CIT0013] Bichet A Desnos T Turner S Grandjean O Hofte H 2001 BOTERO1 is required for normal orientation of cortical microtubules and anisotropic cell expansion in *Arabidopsis* . The Plant Journal 25, 137–148. 11169190 10.1046/j.1365-313x.2001.00946.x

[CIT0014] Blakesley D Weston GD Hall JF 1991 The role of endogenous auxin in root initiation Part I. Evidence from studies on auxin application, and analysis of endogenous levels. Plant Growth and Regulation 10, 341–353.

[CIT0015] Blancaflor EB Hasenstein KH 1995 Time course and auxin sensitivity of cortical microtubule reorientation in maize roots. Protoplasma 185, 72–82. 11541297 10.1007/BF01272755

[CIT0016] Boutte Y Crosnier MT Carraro N Traas J Satiat-Jeunemaitre B 2006 The plasma membrane recycling pathway and cell polarity in plants: studies on PIN proteins. Journal of Cell Science 119, 1255–1265. 16522683 10.1242/jcs.02847

[CIT0017] Braybrook SA Peaucelle A 2013 Mechano-chemical aspects of organ formation in *Arabidopsis thaliana*: the relationship between auxin and pectin. PLoS One 8, e57813. 23554870 10.1371/journal.pone.0057813 PMC3595255

[CIT0018] Bringmann M Li E Sampathkumar A Kocabek T Hauser MT Persson S 2012 POM-POM2/cellulose synthase interacting1 is essential for the functional association of cellulose synthase and microtubules in *Arabidopsis* . The Plant Cell 24, 163–177. 22294619 10.1105/tpc.111.093575 PMC3289571

[CIT0019] Burian A Ludynia M Uyttewaal M Traas J Boudaoud A Hamant O Kwiatkowska D 2013 A correlative microscopy approach relates microtubule behaviour, local organ geometry, and cell growth at the *Arabidopsis* shoot apical meristem. Journal of Experimental Botany 64, 5753–5767. 24153420 10.1093/jxb/ert352 PMC3871827

[CIT0020] Burk DH Liu B Zhong R Morrison WH Ye ZH 2001 A katanin-like protein regulates normal cell wall biosynthesis and cell elongation. The Plant Cell 13, 807–827. 11283338 PMC135546

[CIT0021] Chaimovitsh D Rogovoy Stelmakh O Altshuler O Belausov E Abu-Abied M Rubin B Sadot E Dudai N 2011 The relative effect of citral on mitotic microtubules in wheat roots and BY2 cells. Plant Biology (Stuttg) 14, 354–364. 10.1111/j.1438-8677.2011.00511.x 22039835

[CIT0022] Chen X Grandont L Li H Hauschild R Paque S Abuzeineh A Rakusova H Benkova E Perrot-Rechenmann C Friml J 2014 Inhibition of cell expansion by rapid ABP1-mediated auxin effect on microtubules. Nature 516, 90–93. 25409144 10.1038/nature13889 PMC4257754

[CIT0023] Chen X Naramoto S Robert S Tejos R Lofke C Lin D Yang Z Friml J 2012 ABP1 and ROP6 GTPase signaling regulate clathrin-mediated endocytosis in *Arabidopsis* roots. Current Biology 22, 1326–1332. 22683261 10.1016/j.cub.2012.05.020

[CIT0024] Clough SJ Bent AF 1998 Floral dip: a simplified method for Agrobacterium-mediated transformation of *Arabidopsis thaliana* . The Plant Journal 16, 735–743. 10069079 10.1046/j.1365-313x.1998.00343.x

[CIT0025] Cosgrove DJ 2005 Growth of the plant cell wall. Nature Reviews Molecular Cell Biology 6, 850–861. 16261190 10.1038/nrm1746

[CIT0026] De Klerk GJ Van der Krieken W de Jong JC 1999 The formation of adventitious roots: new concepts, new possibilities. In Vitro Cellular & Developmental Biology - Plant 35, 189–199.

[CIT0027] De Smet I Beeckman T 2011 Asymmetric cell division in land plants and algae: the driving force for differentiation. Nature Reviews Molecular Cell Biology 12, 177–188. 21346731 10.1038/nrm3064

[CIT0028] Dhonukshe P Mathur J Hulskamp M Gadella TW Jr 2005 Microtubule plus-ends reveal essential links between intracellular polarization and localized modulation of endocytosis during division-plane establishment in plant cells. BMC Biology 3, 11. 15831100 10.1186/1741-7007-3-11 PMC1087477

[CIT0029] Ditengou FA Teale WD Kochersperger P 2008 Mechanical induction of lateral root initiation in *Arabidopsis thaliana* . Proceeding of the National Academy of Sciences U S A 105, 18818–18823. 10.1073/pnas.0807814105 PMC2596224 19033199

[CIT0030] Fahn A 1990 Plant Anatomy , Fourth Edition, Pergamon Press, Oxford, UK.

[CIT0031] Falasca G Altmura MM 2003 Histological analysis of adventitious rooting in *Arabidopsis thaliana* (L.) Heynh seedlings. Plant Biosystems 137, 265–274.

[CIT0032] Feraru E Feraru MI Kleine-Vehn J Martiniere A Mouille G Vanneste S Vernhettes S Runions J Friml J 2011 PIN polarity maintenance by the cell wall in *Arabidopsis* . Current Biology 21, 338–343. 21315597 10.1016/j.cub.2011.01.036

[CIT0033] Fisher DD Cyr RJ 1998 Extending the microtubule/microfibril paradigm. Cellulose synthesis is required for normal cortical microtubule alignment in elongating cells. Plant Physiology 116, 1043–1051. 9501137 10.1104/pp.116.3.1043 PMC35074

[CIT0034] Fu Y Gu Y Zheng Z Wasteneys G Yang Z 2005 *Arabidopsis* interdigitating cell growth requires two antagonistic pathways with opposing action on cell morphogenesis. Cell 120, 687–700. 15766531 10.1016/j.cell.2004.12.026

[CIT0035] Fujita M Himmelspach R Hocart CH Williamson RE Mansfield SD Wasteneys GO 2011 Cortical microtubules optimize cell-wall crystallinity to drive unidirectional growth in *Arabidopsis* . The Plant Journal 66, 915–928. 21535258 10.1111/j.1365-313X.2011.04552.x

[CIT0036] Fujita M Himmelspach R Ward J 2013 The anisotropy1 D604N mutation in the *Arabidopsis* cellulose synthase1 catalytic domain reduces cell wall crystallinity and the velocity of cellulose synthase complexes. Plant Physiology 162, 74–85. 23532584 10.1104/pp.112.211565 PMC3641231

[CIT0037] Greenwood MS Cui X Xu F 2001 Response to auxin changes during maturation-related loss of adventitious rooting competence in loblolly pine (*Pinus taeda*) stem cuttings. Physiologia Plantarum 111, 373–380. 11240922 10.1034/j.1399-3054.2001.1110315.x

[CIT0038] Gutierrez L Bussell JD Pacurar DI Schwambach J Pacurar M Bellini C 2009 Phenotypic plasticity of adventitious rooting in *Arabidopsis* is controlled by complex regulation of AUXIN RESPONSE FACTOR transcripts and microRNA abundance. Plant Cell 21, 3119–3132. 19820192 10.1105/tpc.108.064758 PMC2782293

[CIT0039] Hamant O Heisler MG Jonsson H 2008 Developmental patterning by mechanical signals in *Arabidopsis* . Science 322, 1650–1655. 19074340 10.1126/science.1165594

[CIT0040] Hamant O Meyerowitz EM Traas J 2011 Is cell polarity under mechanical control in plants? Plant Signaling and Behavior 6, 137–139. 21258209 10.4161/psb.6.1.14269 PMC3122027

[CIT0041] Hardham AR Green PB Lang JM 1980 Reorganization of cortical microtubules and cellulose deposition during leaf formation in *Graptopetalum paraguayense* . Planta 149, 181–195. 24306251 10.1007/BF00380881

[CIT0042] Heisler MG Hamant O Krupinski P Uyttewaal M Ohno C Jonsson H Traas J Meyerowitz EM 2010 Alignment between PIN1 polarity and microtubule orientation in the shoot apical meristem reveals a tight coupling between morphogenesis and auxin transport. PLoS Biology 8, e1000516. 20976043 10.1371/journal.pbio.1000516 PMC2957402

[CIT0043] Heisler MG Ohno C Das P Sieber P Reddy GV Long JA Meyerowitz EM 2005 Patterns of auxin transport and gene expression during primordium development revealed by live imaging of the *Arabidopsis* inflorescence meristem. Current Biology 15, 1899–1911. 16271866 10.1016/j.cub.2005.09.052

[CIT0044] Hernandez LF Green PB 1993 Transductions for the expression of structural pattern: analysis in sunflower. The Plant Cell 5, 1725–1738. 12271053 10.1105/tpc.5.12.1725 PMC160399

[CIT0045] Himmelspach R Williamson RE Wasteneys GO 2003 Cellulose microfibril alignment recovers from DCB-induced disruption despite microtubule disorganization. The Plant Journal 36, 565–575. 14617086 10.1046/j.1365-313x.2003.01906.x

[CIT0046] Kawamura E Wasteneys GO 2008 MOR1, the *Arabidopsis thaliana* homologue of *Xenopus* MAP215, promotes rapid growth and shrinkage, and suppresses the pausing of microtubules *in vivo* . Journal of Cell Science 121, 4114–4123. 19033380 10.1242/jcs.039065

[CIT0047] Kierzkowski D Nakayama N Routier-Kierzkowska AL Weber A Bayer E Schorderet M Reinhardt D Kuhlemeier C Smith RS 2012 Elastic domains regulate growth and organogenesis in the plant shoot apical meristem. Science 335, 1096–1099. 22383847 10.1126/science.1213100

[CIT0048] Kirik V Herrmann U Parupalli C Sedbrook JC Ehrhardt DW Hulskamp M 2007 CLASP localizes in two discrete patterns on cortical microtubules and is required for cell morphogenesis and cell division in *Arabidopsis* . Journal of Cell Science 120, 4416–4425. 18042620 10.1242/jcs.024950

[CIT0049] Kleine-Vehn J Langowski L Wisniewska J Dhonukshe P Brewer PB Friml J 2008 Cellular and molecular requirements for polar PIN targeting and transcytosis in plants. Molecular Plant 1, 1056–1066. 19825603 10.1093/mp/ssn062

[CIT0050] Konishi M Sugiyama M 2003 Genetic analysis of adventitious root formation with a novel series of temperature-sensitive mutants of *Arabidopsis thaliana* . Development 130, 5637–5647. 14522871 10.1242/dev.00794

[CIT0051] Kumpf RP Shi CL Larrieu A Sto IM Butenko MA Peret B Riiser ES Bennett MJ Aalen RB 2013 Floral organ abscission peptide IDA and its HAE/HSL2 receptors control cell separation during lateral root emergence. Proceeding of the National Academy of Sciences U S A 110, 5235–5240. 10.1073/pnas.1210835110 PMC3612645 23479623

[CIT0052] Landrein B Hamant O 2013 How mechanical stress controls microtubule behavior and morphogenesis in plants: history, experiments and revisited theories. The Plant Journal 7, 324–338. 23551516 10.1111/tpj.12188

[CIT0053] Laskowski M Grieneisen VA Hofhuis H Hove CA Hogeweg P Maree AF Scheres B 2008 Root system architecture from coupling cell shape to auxin transport. PLoS Biology 6, e307. 19090618 10.1371/journal.pbio.0060307 PMC2602721

[CIT0054] Levy A Szwerdszarf D Abu-Abied M Mordehaev I Yaniv Y Riov J Arazi T Sadot E 2014 Profiling microRNAs in *Eucalyptus grandis* reveals no mutual relationship between alterations in miR156 and miR172 expression and adventitious root induction during development. BMC Genomics 15, 524. 24965948 10.1186/1471-2164-15-524 PMC4094776

[CIT0055] Lichtenstein N Geiger B Kam Z 2003 Quantitative analysis of cytoskeletal organization by digital fluorescent microscopy. Cytometry 54A, 8–18. 12820116 10.1002/cyto.a.10053

[CIT0056] Lin D Cao L Zhou Z Zhu L Ehrhardt D Yang Z Fu Y 2013 Rho GTPase signaling activates microtubule severing to promote microtubule ordering in *Arabidopsis* . Current Biology 23, 290–297. 23394835 10.1016/j.cub.2013.01.022

[CIT0057] Lin D Nagawa S Chen J 2012 A ROP GTPase-dependent auxin signaling pathway regulates the subcellular distribution of pin2 in *Arabidopsis* roots. Current Biology 22, 1319–1325. 22683260 10.1016/j.cub.2012.05.019 PMC3407329

[CIT0058] Lin Y Schiefelbein J 2001 Embryonic control of epidermal cell patterning in the root and hypocotyl of *Arabidopsis* . Development 128, 3697–3705. 11585796 10.1242/dev.128.19.3697

[CIT0059] Lindeboom JJ Nakamura M Hibbel A Shundyak K Gutierrez R Ketelaar T Emons AM Mulder BM Kirik V Ehrhardt DW 2013 A mechanism for reorientation of cortical microtubule arrays driven by microtubule severing. Science 342, 1245533. 24200811 10.1126/science.1245533

[CIT0060] Lucas M Kenobi K von Wangenheim D 2013 Lateral root morphogenesis is dependent on the mechanical properties of the overlaying tissues. Proceeding of the National Academy of Sciences U S A 110, 5229–5234. 10.1073/pnas.1210807110 PMC3612681 23479644

[CIT0061] Mei Y Gao HB Yuan M Xue HW 2012 The *Arabidopsis* ARCP protein, CSI1, which is required for microtubule stability, is necessary for root and anther development. The Plant Cell 24, 1066–1080. 22427339 10.1105/tpc.111.095059 PMC3336141

[CIT0062] Murray JA Jones A Godin C Traas J 2012 Systems analysis of shoot apical meristem growth and development: integrating hormonal and mechanical signaling. The Plant Cell 24, 3907–3919. 23110895 10.1105/tpc.112.102194 PMC3517227

[CIT0063] Nagawa S Xu T Lin D Dhonukshe P Zhang X Friml J Scheres B Fu Y Yang Z 2012 ROP GTPase-dependent actin microfilaments promote PIN1 polarization by localized inhibition of clathrin-dependent endocytosis. PLoS Biology 10, e1001299. 22509133 10.1371/journal.pbio.1001299 PMC3317906

[CIT0064] Nakayama N Smith RS Mandel T Robinson S Kimura S Boudaoud A Kuhlemeier C 2012 Mechanical regulation of auxin-mediated growth. Current Biology 22, 1468–1476. 22818916 10.1016/j.cub.2012.06.050

[CIT0065] Oppenoorth JM 1978 The influence of colchicine on initiation and early development of adventitious roots. Physiologia Plantarum 42, 375–378.

[CIT0066] Paredez AR Persson S Ehrhardt DW Somerville CR 2008 Genetic evidence that cellulose synthase activity influences microtubule cortical array organization. Plant Physiology 147, 1723–1734. 18583534 10.1104/pp.108.120196 PMC2492609

[CIT0067] Paredez AR Somerville CR Ehrhardt DW 2006 Visualization of cellulose synthase demonstrates functional association with microtubules. Science 312, 1491–1495. 16627697 10.1126/science.1126551

[CIT0068] Peaucelle A Braybrook SA Le Guillou L Bron E Kuhlemeier C Hofte H 2011 Pectin-induced changes in cell wall mechanics underlie organ initiation in *Arabidopsis* . Current Biology 21, 1720–1726. 21982593 10.1016/j.cub.2011.08.057

[CIT0069] Pien S Wyrzykowska J McQueen-Mason S Smart C Fleming A 2001 Local expression of expansin induces the entire process of leaf development and modifies leaf shape. Proceeding of the National Academy of Sciences U S A 98, 11812–11817. 10.1073/pnas.191380498 PMC58813 11562463

[CIT0070] Rasmussen A Mason MG 2012 Strigolactones suppress adventitious rooting in *Arabidopsis* and pea. Plant Physiology 158, 1976–1987. 22323776 10.1104/pp.111.187104 PMC3320200

[CIT0071] Rasmussen CG Humphries JA Smith LG 2011 Determination of symmetric and asymmetric division planes in plant cells. Annual Reviews Plant Biology 62, 387–409. 10.1146/annurev-arplant-042110-103802 21391814

[CIT0072] Rayle DL Cleland RE 1992 The Acid Growth Theory of auxin-induced cell elongation is alive and well. Plant Physiology 99, 1271–1274. 11537886 10.1104/pp.99.4.1271 PMC1080619

[CIT0073] Richter GL Monshausen GB Krol A Gilroy S 2009 Mechanical stimuli modulate lateral root organogenesis. Plant Physiology 151, 1855–1866. 19794120 10.1104/pp.109.142448 PMC2785988

[CIT0074] Riov J Szwerdszarf D Abu-Abied M Sadot E 2013 The molecular mechanisms involved in adventitious root formation. In Eshel A Beeckman T, eds, Plant Roots: The Hidden Half , Ed 4 Taylor & Francis, London, UK, pp 11.11–11.14.

[CIT0075] Sampathkumar A Krupinski P Wightman R Milani P Berquand A Boudaoud A Hamant O Jonsson H Meyerowitz EM 2014 Subcellular and supracellular mechanical stress prescribes cytoskeleton behavior in *Arabidopsis* cotyledon pavement cells. Elife 3, e01967. 24740969 10.7554/eLife.01967 PMC3985187

[CIT0076] Sassi M Ali O Boudon F Cloarec G 2014 An auxin-mediated shift toward growth isotropy promotes organ formation at the shoot meristem in *Arabidopsis* . Current Biology 24, 2335–2342. 25264254 10.1016/j.cub.2014.08.036

[CIT0077] Stavang JA Gallego-Bartolome J Gomez MD Yoshida S Asami T Olsen JE Garcia-Martinez JL Alabadi D Blazquez MA 2009 Hormonal regulation of temperature-induced growth in *Arabidopsis* . The Plant Journal 60, 589–601. 19686536 10.1111/j.1365-313X.2009.03983.x

[CIT0078] Steffens B Kovalev A Gorb SN Sauter M 2012 Emerging roots alter epidermal cell fate through mechanical and reactive oxygen species signaling. The Plant Cell 24, 3296–3306. 22904148 10.1105/tpc.112.101790 PMC3462632

[CIT0079] Sugimoto K Himmelspach R Williamson RE Wasteneys GO 2003 Mutation or drug-dependent microtubule disruption causes radial swelling without altering parallel cellulose microfibril deposition in *Arabidopsis* root cells. The Plant Cell 15, 1414–1429. 12782733 10.1105/tpc.011593 PMC156376

[CIT0080] Sugimoto K Jiao Y Meyerowitz EM 2010 *Arabidopsis* regeneration from multiple tissues occurs via a root development pathway. Developmental Cell 18, 463–471. 20230752 10.1016/j.devcel.2010.02.004

[CIT0081] Swarup K Benkova E Swarup R 2008 The auxin influx carrier LAX3 promotes lateral root emergence. Nature Cell Biology 10, 946–954. 18622388 10.1038/ncb1754

[CIT0082] Uyttewaal M Burian A Alim K 2012 Mechanical stress acts via katanin to amplify differences in growth rate between adjacent cells in *Arabidopsis* . Cell 149, 439–451. 22500806 10.1016/j.cell.2012.02.048

[CIT0083] Vermeer JE von Wangenheim D Barberon M Lee Y Stelzer EH Maizel A Geldner N 2014 A spatial accommodation by neighboring cells is required for organ initiation in *Arabidopsis* . Science 343, 178–183. 24408432 10.1126/science.1245871

[CIT0084] Vidal N Arellano G San-Jose MC Vieitez AM Ballester A 2003 Developmental stages during the rooting of *in-vitro*-cultured *Quercus robur* shoots from material of juvenile and mature origin. Tree Physiology 23, 1247–1254. 14652224 10.1093/treephys/23.18.1247

[CIT0085] Wasteneys GO 2004 Progress in understanding the role of microtubules in plant cells. Current Opinion Plant Biology 7, 651–660. 10.1016/j.pbi.2004.09.008 15491913

[CIT0086] Wasteneys GO 2002 Microtubule organization in the green kingdom: chaos or self-order? Journal of Cell Science 115, 1345–1354. 11896182 10.1242/jcs.115.7.1345

[CIT0087] Wasteneys GO Collings DA 2004 Expanding beyond the great divide: the cytoskeleton and axial growth. In Hussey PJ, ed, The Plant and Cytoskeleton in Cell Differentiation and Development , Vol. 10 Blackwell Publishing, Oxford, UK, pp 83–116.

[CIT0088] Wasteneys GO Fujita M 2006 Establishing and maintaining axial growth: wall mechanical properties and the cytoskeleton. Journal of Plant Research 119, 5–10. 16284708 10.1007/s10265-005-0233-3

[CIT0089] Whittington AT Vugrek O Wei KJ Hasenbein NG Sugimoto K Rashbrooke MC Wasteneys GO 2001 MOR1 is essential for organizing cortical microtubules in plants. Nature 411, 610–613. 11385579 10.1038/35079128

[CIT0090] Williamson RE 1990 Alignment of cortical microtubules by anisotropic wall stresses. Australian Journal of Plant Physiology 17, 601–613.

[CIT0091] Wymer CL Wymer SA Cosgrove DJ Cyr RJ 1996 Plant cell growth responds to external forces and the response requires intact microtubules. Plant Physiology 110, 425–430. 11536739 10.1104/pp.110.2.425 PMC157736

[CIT0092] Xu T Dai N Chen J 2014 Cell surface ABP1-TMK auxin-sensing complex activates ROP GTPase signaling. Science 343, 1025–1028. 24578577 10.1126/science.1245125 PMC4166562

[CIT0093] Zandomeni K Schopfer P 1994 Mechanosensory microtubule reorientation in the epidermis of maize coleoptiles subjected to bending stress. Protoplasma 182, 96–101. 11540618 10.1007/BF01403471

[CIT0094] Zhang Q Fishel E Bertroche T Dixit R 2013 Microtubule severing at crossover sites by katanin generates ordered cortical microtubule arrays in *Arabidopsis* . Current Biology 23, 2191–2195. 24206847 10.1016/j.cub.2013.09.018

