# Supplementary material for: Dissecting the contribution of microtubule behaviour in adventitious root induction
Source: J Exp Bot. 2015 Mar 18;66(9):2813–24. doi: 10.1093/jxb/erv097 (PMC4986881; doi:10.1093/jxb/erv097)
Supplement: Supplementary Data [file supp_66_9_2813__index.html]

Dissecting the contribution of microtubule behaviour in adventitious root induction — Dissecting the contribution of microtubule behaviour in adventitious root induction — Supplementary Data 

# Dissecting the contribution of microtubule behaviour in adventitious root induction

## Supplementary Data

Data files

**Files in this Data Supplement:**

- Supplementary Data - Supplementary Data
- Supplementary Data - Supplementary Data
- Supplementary Data - Supplementary Data
- Supplementary Data - Supplementary Data
- Supplementary Data - Supplementary Data
- Supplementary Data - Supplementary Data
- Supplementary Data - Supplementary Data
