# Supplementary material for: Dissecting the contribution of microtubule behaviour in adventitious root induction
Source: J Exp Bot. 2015 Mar 18;66(9):2813–24. doi: 10.1093/jxb/erv097 (PMC4986881; doi:10.1093/jxb/erv097)
Supplement: Supplementary Data [file supp_erv097_jexbot142422_file007.pdf]

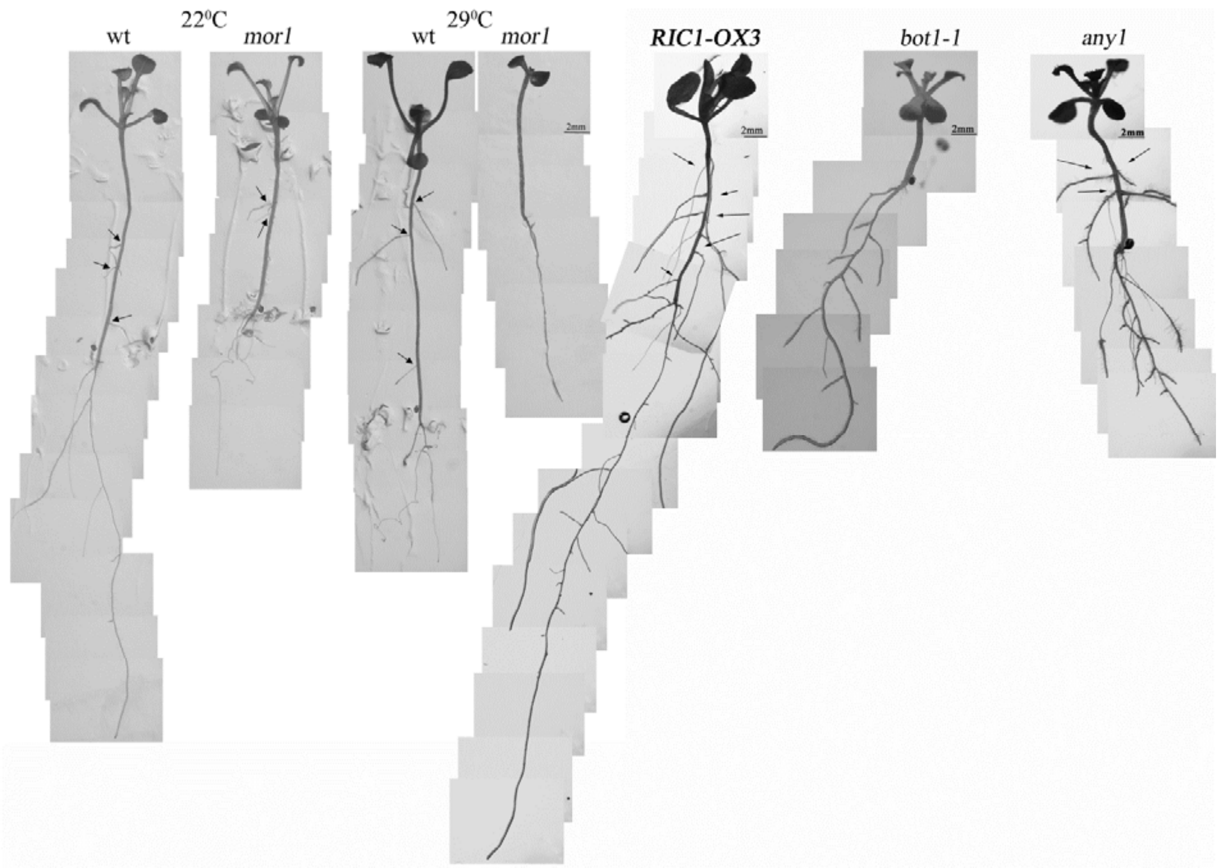

Supplementary Figure 1. The different mutant plants that were induced to form AR by the dark to light regime. A representative plant was imaged after 14 days. All scale bars are 2mm. Arrows show ARs.

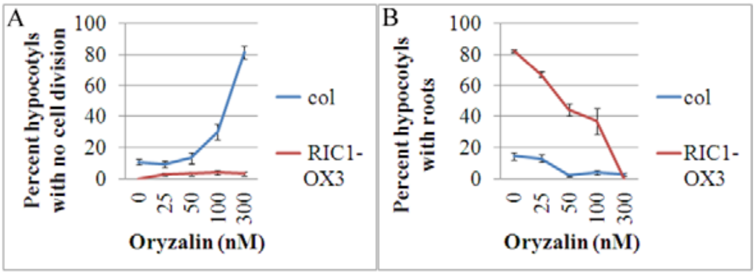

Supplementary Figure 2. Hypocotyls (5-6mm in length) were excised from etiolated seedlings of either wild type plants or RIC1-OX3 plants and incubated in MS with 1% sucrose,10μM IBA and increasing amounts of oryzalin for 3 days. Quantitative analysis of three biological replications was performed. A . Percent hypocotyls with no detectable cell division. B. percent hypocotyls with roots.

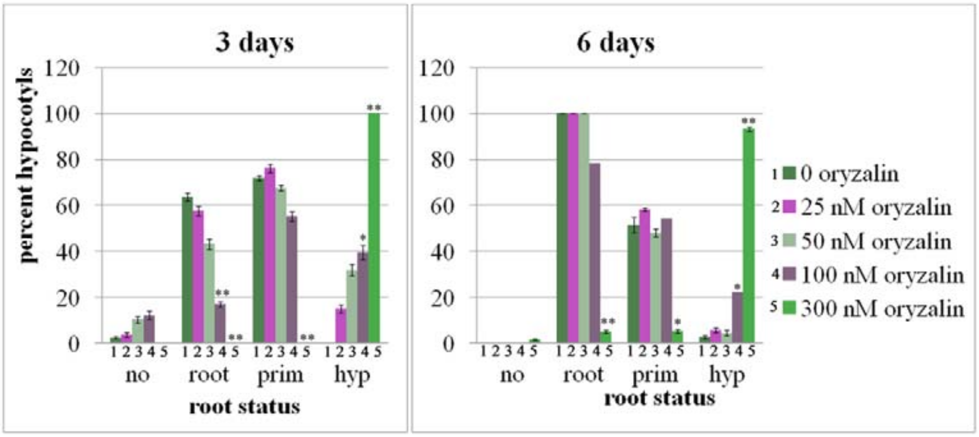

Supplementary Figure 3. Hypocotyls (5-6mm in length) were excised from etiolated seedlings and incubated in MS with 1% sucrose,10μM IBA and increasing concentrations of oryzalin for 3 or 6 days. Quantitative analysis of three biological replications was performed to determine the percentage of hypocotyls without roots (NO), primordia (prim), roots or hyperplasia (hyp). Asterisks show significant difference from control (O nM oryzalin) as determined by Scheffe analysis *p<0.05, **p<0.01.

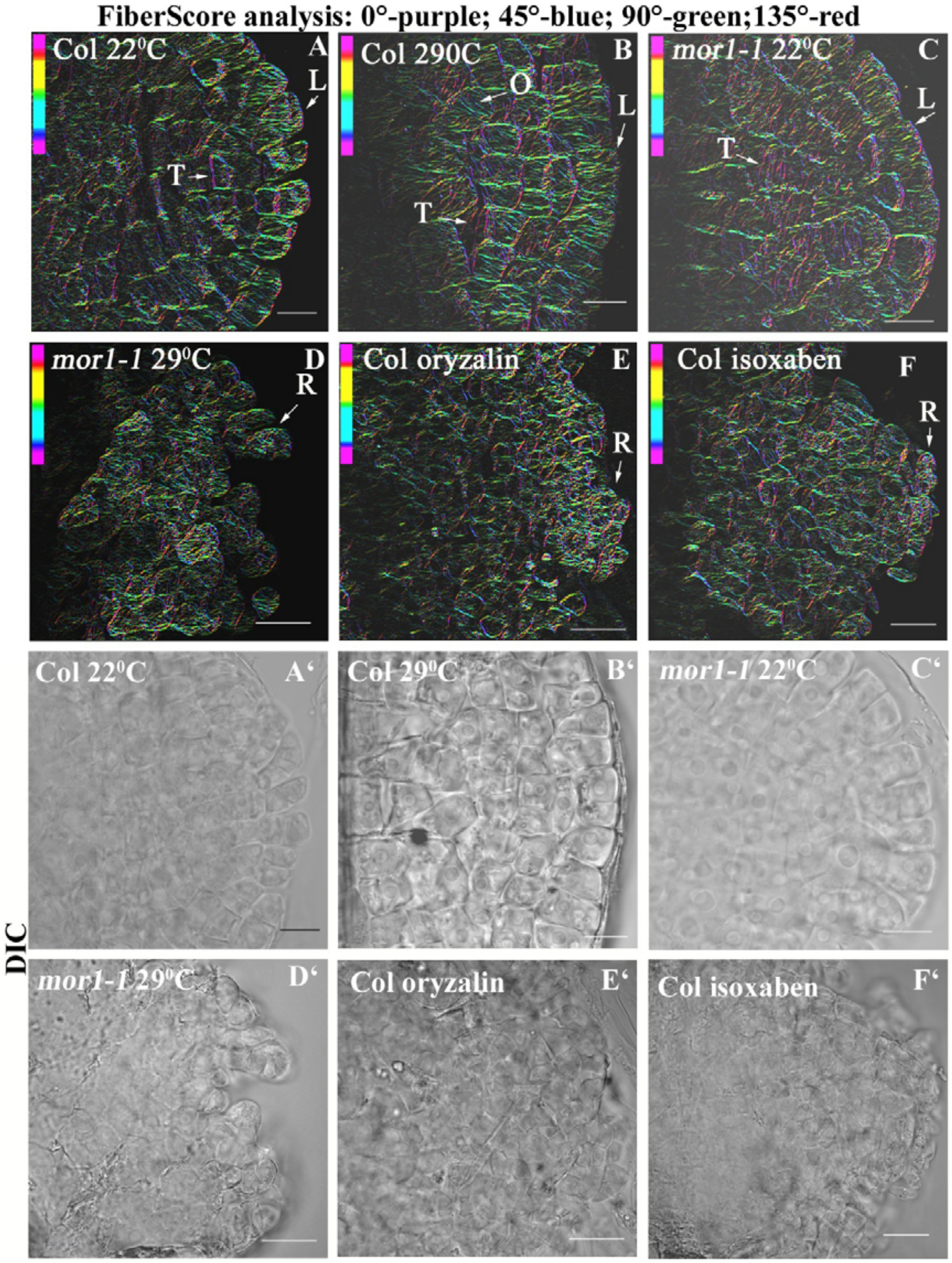

Supplementary Figure 4. Stage V primordia or clusters of similar size were stained for MTs. MTs were analysed by FiberScore after filtering with the rolling ball filter. (A-F) show a MTs colored orientation map for representative primordia or cluster. (A'-F') are corresponding DIC images. Scale bars in A-C, and A'-C' are 10μm, and in D-F and D'-F' are 20μm. T=transverse. L= longitudinal, O= oblique, R= random.

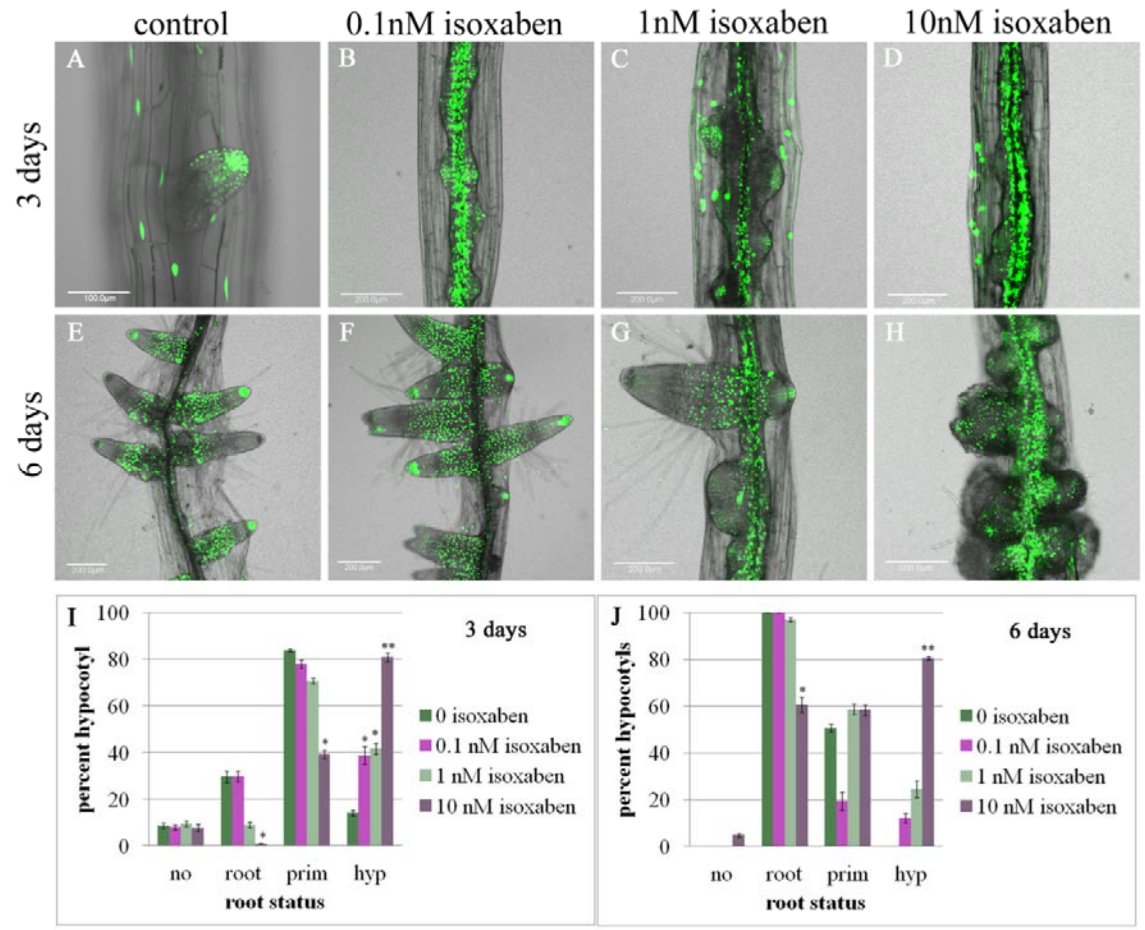

Supplementary Figure 5. Hypocotyls (5-6mm in length) were excised from etiolated seedlings and incubated in MS with 1% sucrose,10µM IBA and increasing amounts of isoxaben for 3 (A-D) or 6 (E-H) days. (I, J) Quantitative analysis of three biological replications was performed of the amount of hypocotyls without roots (NO), primordia (prim), roots or hyperplasia (hyp). Asterisks show significant difference from control (O nM isoxaben) as determined by Scheffe analysis $^*p<0.05$, $^{**}p<0.01$.

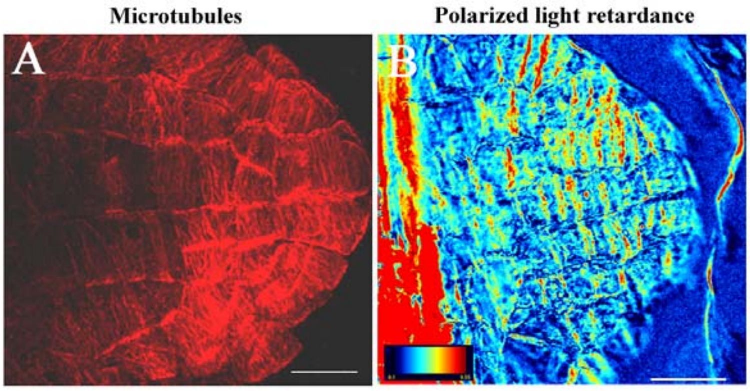

Supplementary Figure 6. A comparison of MT orientation and polarized light retardance pattern in epidermal cells of AR primordia at stage V. (A). Stage V primordia stained for MT and imaged by a confocal microscope. Shown is a projection of several optic sections, scale bar is 20 µm. (B). A 10µm section of a stage V AR primordia, made by a rotary microtome, imaged by a PoleScope and analysed by the Abrio software of Scale bar is 30µm. The colored scale is from blue (0 nm) to red (10 nm) wave lengths

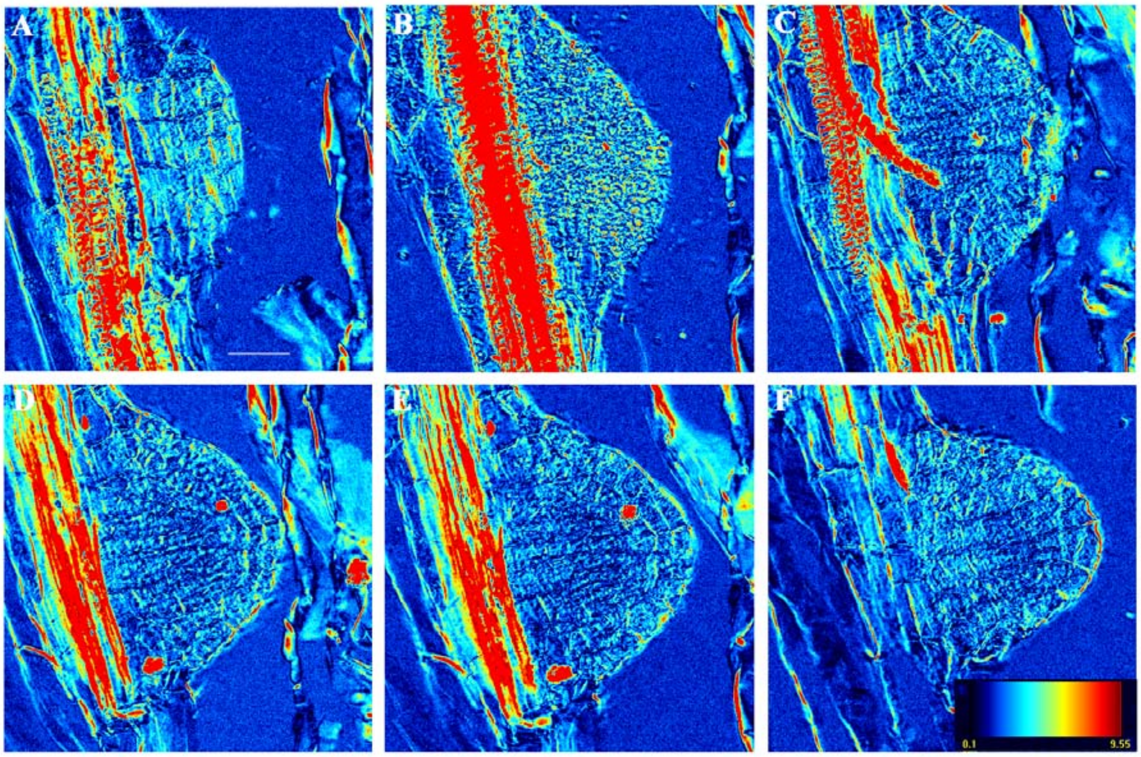

Supplementary Figure 7. (A-F) Serial 10µm sections through a stage V AR primordia, imaged by the PoleScope. The typical high light reterdance (red) that is created by the hypocotyl xylem cell walls is absent in the AR primordia. The colored scale is from blue (0 nm) to red (10 nm) wave lengths. Scale bar is 30µm.

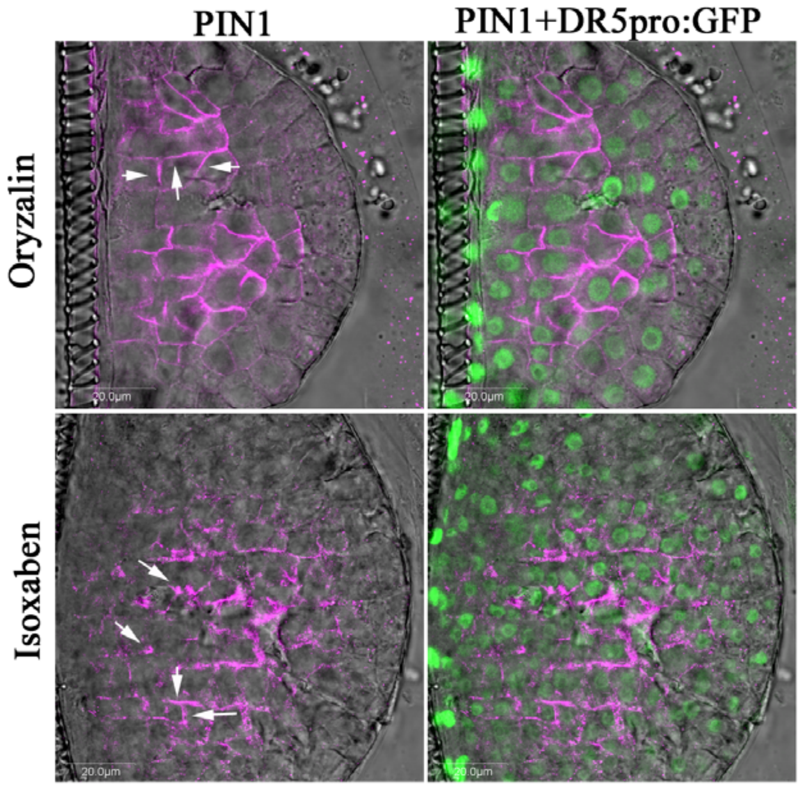

Supplementary figure 8. Close-up on the distribution of PIN1 in the presence of oryzalin or isoxaben. Arrows show localization at more than one cell face in both cases and at 3-way cell junctions in the presence of isoxaben. Scale bars are 20µm.

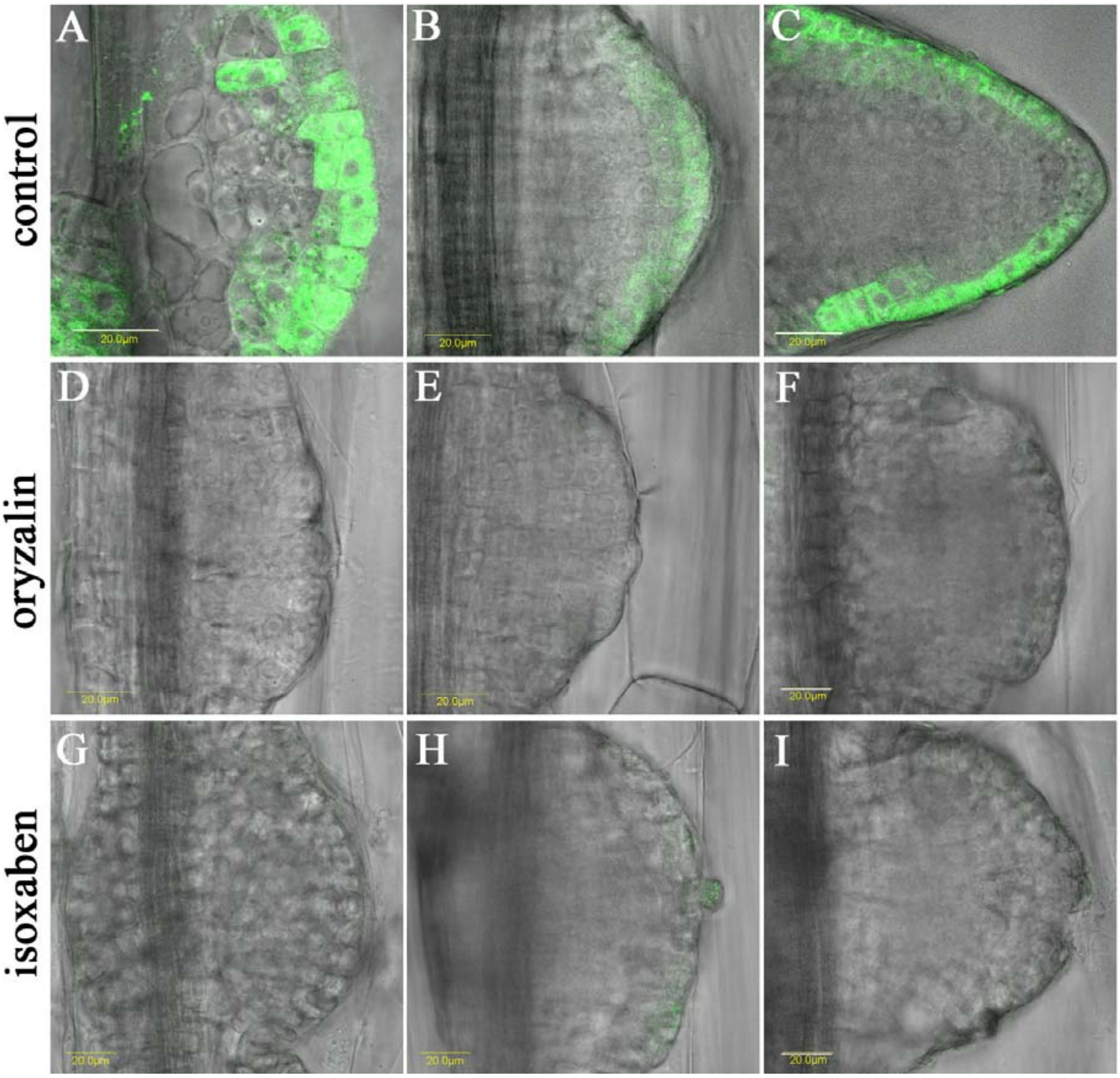

Supplementary Figure 9. AR primordia of different stages of plants expressing the $GL2_{prom}$:GFP. (A-C) Primirdia of stages IV, V and VI respectively. (D-F) Cell clusters at different sizes, which were created in the presence of 100 nM of oryzalin. (G-I) Cell clusters at different sizes, which were created in the presence of 10 nM of isoxaben. All images were acquired 3 days post induction. Scale bars are 20μm.
